# Numerical Solutions of the Nonlinear Fractional-Order Brusselator System by Bernstein Polynomials

**DOI:** 10.1155/2014/257484

**Published:** 2014-11-17

**Authors:** Hasib Khan, Hossein Jafari, Rahmat Ali Khan, Haleh Tajadodi, Sarah Jane Johnston

**Affiliations:** ^1^Department of Mathematics, University of Malakand, Dir Lower, Khyber Pakhtunkhwa 18000, Pakistan; ^2^Shaheed Benazir Bhutto University, Sheringal, Dir Upper, Khyber Pakhtunkhwa 18000, Pakistan; ^3^Department of Mathematical Sciences, University of South Africa, P.O. Box 392, UNISA 0003, South Africa; ^4^Department of Mathematics, University of Mazandaran, P.O. Box 47416-95447, Babolsar, Iran

## Abstract

In this paper we propose the Bernstein polynomials to achieve the numerical solutions of nonlinear fractional-order chaotic system known by fractional-order Brusselator system. We use operational matrices of fractional integration and multiplication of Bernstein polynomials, which turns the nonlinear fractional-order Brusselator system to a system of algebraic equations. Two illustrative examples are given in order to demonstrate the accuracy and simplicity of the proposed techniques.

## 1. Introduction

Fractional calculus has applications in many scientific disciplines based on mathematical modeling including signal and image processing, physics, aerodynamics, chemistry, economics, electrodynamics, polymer rheology, economics, biophysics, control theory, and blood flow phenomena (cf. [1–7]). Researchers are investigating and developing fractional calculus in different ways including the numerical solutions of fractional-order differential equations using different numerical tools. There is interesting and valuable work in the literature for the numerical solutions of fractional-order differential equations using Bernstein polynomials (BPs). This work has interested many researchers recently (see, e.g., [8–13]).

Chaos theory is considered an important tool for viewing and understanding our universe and different techniques are utilized in order to reduce problems produced by the unusual behaviours of chaotic systems including chaos control (cf. [14, 15]). In the literature, several authors have considered the chaotic system known as the fractional-order Brusselator system (FOBS) recently (cf. [7, 16]). For example, Gafiychuk and Datsko investigate the stability of fractional-order Brusselator system in [17]. In [18], Wang and Li proved that the solution of fractional-order Brusselator system has a limit cycle using numerical method. Jafari et al. used the variational iteration method to investigate the approximate solutions of this system [19].

In this paper, we are interested in obtaining the numerical solution of the nonlinear fractional-order Brusselator system given by

(1) $$\begin{array}{c} D_{t}^{\alpha }x\left(t\right)=a-\left(\mu +1\right)x\left(t\right)+x^{2}\left(t\right)y\left(t\right),\\ D_{t}^{\beta }y\left(t\right)=\mu x\left(t\right)-x^{2}\left(t\right)y\left(t\right), \end{array}$$

with initial conditions

(2) $$\begin{array}{c} x\left(0\right)=c_{1},\;\;y\left(0\right)=c_{2} \end{array}$$

by means of operational matrices of fractional-order integration and multiplication of Bernstein polynomials, provided that *a* > 0, *μ* > 0, *α*, *β* ∈ (0,1], and *c*_1_, *c*_2_ are constants. Moreover, *D* ^*α*^, *D* ^*β*^ represent Caputo's derivative of order *α*, *β*, respectively [1, 6], namely,

(3) Dtαf(t) =1Γ(n−α)∫0tf(τ)t−τ1+α−n dτ,n−1<α<n,  n∈Ndndtnf(x),α=n.

Note that

(4)  i  DtαC=0, (C  is  a  constant),

(5) ii  Dtαtβ   =0β∈N,  β<αΓ(β+1)Γ(1+β−α)tβ−α,β∈N,  β≥α  orβ∉N,  β>α,

(6)  iii  ItαDtαft=ft−∑k=0n−1fk0+tkk!, n−1<α≤n,

where *I*_*t*_^*α*^ denotes the fractional Riemann-Liouville integral [1, 6], namely,

(7) $$\begin{array}{c} I_{t}^{\alpha }f\left(t\right)=\frac{1}{\Gamma \left(\alpha \right)}\overset{t}{\underset{0}{\int }}\frac{f\left(\tau \right)}{{\left(t-\tau \right)}^{1-\alpha }}\;d\tau ,\;\alpha >0. \end{array}$$

Detailed explanations regarding the properties of the fractional operators may be found in [1, 6].

In Section 2, we discuss the Bernstein polynomials and their properties. Also, we give the approximation of functions via Bernstein polynomials. In Section 3, we discuss operational matrices for fractional integration and multiplication via Bernstein polynomials. In Section 4, we give a numerical scheme for the Brusselator system based on Bernstein polynomials. In Section 5, illustrative examples are given which demonstrate the accuracy of our scheme based on the operational matrices for fractional-order integration of Bernstein polynomials. In the final section, a summary of the paper is presented.

## 2. Bernstein Polynomials and Their Properties

### 2.1. Definition of Bernstein Polynomials

We consider the Bernstein polynomials of the *m*th degree on the interval on [0,1] (cf. [11]) given by

(8) $$\begin{array}{c} B_{i,m}\left(t\right)=\left(\begin{array}{c} m\\ i \end{array}\right)t^{i}{\left(1-t\right)}^{m-i},\;0\leq i\leq m. \end{array}$$

The Bernstein polynomials satisfy the recursive definition given by

(9) $$\begin{array}{c} B_{i,m}\left(t\right)=\left(1-t\right)B_{i,m-1}\left(t\right)+tB_{i-1,m-1}\left(t\right),\;i=0,1\ldots ,m. \end{array}$$

By using the binomial expansion of (1 − *t*)^*m*−*i*^, Bernstein polynomials can be shown in terms of linear combination of the basis functions:

(10) Bi,mt=miti1−tm−i=miti∑k=0m−i−1km−iktk=∑k=0m−i−1kmim−ikti+k, i=0,1,…,m.

We can write the Bernstein polynomials in the form *B*_*i*,*m*_(*t*) = *A*_*i*+1_ *T*_*m*_(*t*), for *i* = 0,1,…, *m*, where

(11) Ai+1=0,0,…,0,−10mi,−11mim−i1,…,  −1m−imim−im−i,Tm(x)=1t⋮tm.

Now if we introduce (*m* + 1)×(*m* + 1) matrix *A* in the form

(12) $$\begin{array}{c} A=\left[\begin{array}{c} A_{1}\\ A_{2}\\ \vdots \\ A_{m+1} \end{array}\right], \end{array}$$

then we have *ϕ*(*t*) = *AT*_*m*_(*t*), where *ϕ*(*t*) = [*B*_0,*m*_(*t*), *B*_1,*m*_(*t*),…,*B*_*m*,*m*_(*t*)]^*T*^ and matrix *A* is an upper triangular matrix given by

(13) $$\begin{array}{c} A=\left[\begin{array}{cccc} {\left(-1\right)}^{0}\left(\begin{array}{c} m\\ 0 \end{array}\right) & {\left(-1\right)}^{1}\left(\begin{array}{c} m\\ 0 \end{array}\right)\left(\begin{array}{c} m-0\\ 1-0 \end{array}\right) & \cdots & {\left(-1\right)}^{m-0}\left(\begin{array}{c} m\\ 0 \end{array}\right)\left(\begin{array}{c} m-0\\ m-0 \end{array}\right)\\ \ddots & & & \vdots \\ 0 & {\left(-1\right)}^{0}\left(\begin{array}{c} m\\ i \end{array}\right) & \cdots & {\left(-1\right)}^{m-i}\left(\begin{array}{c} m\\ i \end{array}\right)\left(\begin{array}{c} m-i\\ m-i \end{array}\right)\\ \vdots & \ddots & \ddots & \vdots \\ 0 & \cdots & 0 & {\left(-1\right)}^{m}\left(\begin{array}{c} m\\ m \end{array}\right) \end{array}\right], \end{array}$$

where $\left|A\right|=\Pi_{i=0}^{m}\left(\begin{array}{c} m\\ i \end{array}\right)$. Thus *A* is an invertible matrix.

### 2.2. Approximation of Function

The set of Bernstein polynomials {*B*_0,*m*_, *B*_1,*m*_,…, *B*_*m*,*m*_} in Hilbert space *L* ^2^[0,1] is a complete basis (cf. [20]). Therefore, any function can be represented by Bernstein polynomials by means of

(14) $$\begin{array}{c} f\left(t\right)=\overset{m}{\underset{i=0}{\sum }}c_{i}B_{i,m}=C^{T}\phi , \end{array}$$

where *ϕ* ^*T*^ = [*B*_0,*m*_, *B*_1,*m*_,…, *B*_*m*,*m*_] and *c* ^*T*^ = [*c*_0_, *c*_1_,…, *c*_*m*_]. Then *c* ^*T*^ can be obtained by

(15) $$\begin{array}{c} C^{T}\langle \phi ,\phi \rangle =\langle f,\phi \rangle , \end{array}$$

where

(16) f,ϕ=∫01f(t)ϕtTdt=[〈f,B0,m〉,〈f,B1,m〉,…,〈f,Bm,m〉],

and 〈*ϕ*, *ϕ*〉 is called dual matrix of *ϕ* which is showed by *Q* where

(17) $$\begin{array}{c} Q=\langle \phi ,\phi \rangle =\overset{1}{\underset{0}{\int }}\phi \left(t\right)\phi {\left(t\right)}^{T}dt. \end{array}$$

Thus

(18) $$\begin{array}{c} C^{T}=\left(\overset{1}{\underset{0}{\int }}f\left(t\right)\phi {\left(t\right)}^{T}dt\right)Q^{-1}, \end{array}$$

where *Q* is the symmetric (*m* + 1)×(*m* + 1) matrix where

(19) Qi+1,j+1=∫01Bi,m(t)Bj,m(t)dt=ninj∫011−t2n−(i+j)ti+jdt=ninj2n+12ni+j i,j=0,1,…,m.

**Lemma 1 .**

Suppose that the function *y* : [0,1] → *R* is (*m* + 1)-times continuously differentiable, and *S*_*m*_ = *Span*⁡{*B*_0,*m*_, *B*_1,*m*_,…, *B*_*m*,*m*_}. If *C* ^*T*^ *ϕ* is the best approximation *y* out of  *S*_*m*_ then

(20) $$\begin{array}{c} {\left\|y-C^{T}\phi \right\|}_{L^{2}[0,1]}\leq \frac{\hat{k}}{\left(m+1\right)!\sqrt{2m+3}}, \end{array}$$

where $\hat{k}=\max_{t\in [0,1]}\left|f^{m+1}(t)\right|$.

**Proof**

See [9].

## 3. Operational Matrix of Bernstein Polynomials

### 3.1. Operational Matrix for Fractional Integration Based on Bernstein Polynomials

The operational matrices of fractional integration of the vector Φ(*t*) can be approximated (cf. [21]) as follows:

(21) Itα0ϕ(t)≃Iαϕ(t),

where *I* ^*α*^ is the (*m* + 1)×(*m* + 1) Riemann-Liouville fractional operational matrix of integration for Bernstein polynomials. By the use of (7), we have

(22) Itα0ϕ(t)=1Γ(α)∫0tt−τα−1ϕm(τ)dτ=1Γ(α)tα−1∗ϕ(t),

where the operator ∗ denotes the convolution product. By substituting *ϕ*(*t*) = *AT*_*m*_(*t*) and from (5) we get

(23) 1Γ(α)tα−1∗ϕ(t) =1Γ(α)tα−1∗(ATm(t))=1Γ(α)A(tα−1∗Tm(t)) =AΓ(α)tα−1∗1,tα−1∗t,…,tα−1∗tT =AIα1,Iαt,…,IαtmT =A0!Γ(α+1)tα,1!Γ(α+2)tα+1,…,m!Γ(α+m+1)tα+mT =ADT¯m,

where *D* is (*m* + 1)×(*m* + 1) matrix and *D* and ${\bar{T}}_{m}$ are given by

(24) $$\begin{array}{c} D=\left[\begin{array}{cccc} \frac{0!}{\Gamma \left(\alpha +1\right)} & 0 & \cdots & 0\\ 0 & \frac{1!}{\Gamma \left(\alpha +2\right)} & \cdots & 0\\ \vdots & \vdots & \ddots & \vdots \\ 0 & 0 & \cdots & \frac{m!}{\Gamma \left(\alpha +m+1\right)} \end{array}\right],\\ {\bar{T}}_{m}=\left[\begin{array}{c} t^{\alpha }\\ t^{\alpha +1}\\ \vdots \\ t^{\alpha +m} \end{array}\right]. \end{array}$$

Now we approximate *t* ^*k*+*α*^ by *m* + 1 terms of the Bernstein basis:

(25) $$\begin{array}{c} t^{\alpha +i}≃E_{i}^{T}\phi_{m}\left(t\right). \end{array}$$

We have

(26) Ei=Q−1∫01tα+iϕ(t)dt=Q−1∫01tα+iB0,m(t)dt,∫01tα+iB1,m(t)dt,…,   ih∫01tα+iBm,m(t)dtT=Q−1E¯i,

where ${\bar{E}}_{i}=[{\bar{E}}_{i,0},{\bar{E}}_{i,1},\ldots ,{\bar{E}}_{i,m}]$ and

(27) E¯i,j=∫01tα+iBi,jtdt=m!Γi+j+α+1j!Γi+m+α+2,hhhhhi,j=0,1,…,m.

Then *E* is (*m* + 1)×(*m* + 1) matrix that has vector $Q^{-1}{\bar{E}}_{i}$ for *i*th columns. Therefore, we can write

(28) Iαϕt=ADE0Tϕt,E1Tϕt,…,EmTϕtT=ADETϕ(t).

Finally, we obtain

(29) Itα0ϕt≃Iαϕt,

where

(30) $$\begin{array}{c} I^{\alpha }=ADE \end{array}$$

is called fractional integration within the operational matrix.

### 3.2. Operational Matrix of Multiplication

It is always necessary to assess the product of *ϕ*(*t*) and *ϕ*(*t*)^*T*^, which is called the product matrix for the Bernstein polynomial basis. The operational matrices for the product $\hat{C}$ are given by

(31) $$\begin{array}{c} C^{T}\phi \left(t\right)\phi {\left(t\right)}^{T}≃\phi {\left(t\right)}^{T}\hat{C}, \end{array}$$

where $\hat{C}$ is (*m* + 1)×(*m* + 1) matrix. So we have

(32) CTϕ(t)ϕtT =CTϕ(t)TmtTAT =[CTϕ(t),t(CTϕm(t)),…,tm(CTϕm(t))]AT =∑i=0nciBi,m(t),∑i=0ncitBi,m(t),…,∑i=0ncitmBi,m(t).

Now, we approximate all functions *t* ^*k*^ *B*_*i*,*n*_(*t*) in terms of {*B*_*i*,*m*_}_*i*=0_^*m*^ for *i*, *k* = 0,1,…, *m*. From (14), we have

(33) $$\begin{array}{c} t^{k}B_{i,m}\left(t\right)≃e_{k,i}^{T}\phi_{m}\left(t\right), \end{array}$$

where *e*_*k*,*i*_ = [*e*_*k*,*i*_^0^, *e*_*k*,*i*_^1^,…, *e*_*k*,*i*_^*m*^]^*T*^. Then we obtain the components of the vector of *e*_*k*,*i*_ where

(34) ek,ij=Q−1∫01tkBi,m(t)ϕ(t)dt=Q−1∫01tkBi,m(t)B0,m(t)dt,   ih∫01tkBi,m(t)B1,m(t)dt,…,tkBi,mtBm,mtdtT=Q−12m+k+1m02m+ki+k,m12m+ki+k+1,…,mm2m+ki+k+mT                hhhhhhhi,k=0,1,…,m.

Thus we obtain

(35) ∑i=0ncitkBi,m(t) =∑i=0nci∑j=0nek,ijBj,m(t)=∑j=0nBj,m(t)∑i=0nciek,ij =ϕmtT∑i=0nciek,i0,∑i=0nciek,i1,…,∑i=0nciek,imT =ϕmtTek,0,ek,1,…,ek,mC=ϕmtTVk+1C,

where *V*_*k*+1_  (*k* = 0,1 …, *m*) is an (*m* + 1)×(*m* + 1) matrix that has vectors *e*_*k*,*i*_  (*i* = 0,1,…, *m*) given for each column. If we choose an (*m* + 1)×(*m* + 1) matrix $\overset{-}{C}=[V_{1}c,V_{1}c,\ldots ,V_{m+1}c]$, then from (32) and (35) we can write

(36) $$\begin{array}{c} C^{T}\phi \left(t\right)\phi {\left(t\right)}^{T}≃\phi {\left(t\right)}^{T}\overset{-}{C}A^{T}, \end{array}$$

and therefore we obtain the operational matrix of product, $\hat{C}=\overset{-}{C}A^{T}$.

**Corollary 2 .**

If *y*(*t*) = *C* ^*T*^ *ϕ*(*t*), consequently one can get the approximate function for *y* ^*k*^(*t*), using Bernstein polynomials by

(37) $$\begin{array}{c} y^{k}\left(t\right)=\phi {\left(t\right)}^{T}{\tilde{C}}_{k}, \end{array}$$

where ${\tilde{C}}_{k}={\hat{C}}^{k-1}C$ and $\hat{C}$ is (*m* + 1)×(*m* + 1) operational matrix of product using Bernstein polynomials.

**Proof**

This arises obviously from [8].

## 4. Numerical Solution of Nonlinear Fractional-Order Brusselator Systems Using Bernstein Polynomials

In this paper, we employ the Bernstein polynomials for solving the nonlinear fractional-order Brusselator systems given in (1). Firstly, we expand the fractional derivative in (1) by the Bernstein basis *ϕ* as follows. Taking

(38) $$\begin{array}{c} D^{\alpha }x\left(t\right)=K^{T}\phi \left(t\right), \end{array}$$

where

(39) $$\begin{array}{c} K^{T}=\left[k_{0},k_{1},\ldots ,k_{m}\right],\\ \phi^{T}=\left[B_{0,m},B_{1,m},\ldots ,B_{m,m}\right], \end{array}$$

are unknowns, and using initial conditions (2), (6), and (30), we approximate *x*(*t*) by

(40) x(t)=Itα0Dtαx(t)+x(0)≈(KTIα+dT)ϕ(t)=GαTϕ(t),

where (*K* ^*T*^ *I* ^*α*^ + *d* ^*T*^) = *G*_*α*_^*T*^ and *I* ^*α*^ is the fractional operational matrix of integration of order *α* and

(41) $$\begin{array}{c} d^{T}=\left[x_{0},x_{0},\ldots ,x_{0}\right]. \end{array}$$

Similarly, we approximate *y*(*t*) from (1) by Bernstein polynomials as

(42) y(t)=Itβ0Dtβx(t)+y(0)≈(RTIβ+d2T)ϕ(t)=HβTϕ(t),

where (*R* ^*T*^ *I* ^*β*^ + *d* ^*T*^) = *H*_*β*_^*T*^ and *I* ^*β*^ is the fractional operational matrix of integration of order *β* and

(43) $$\begin{array}{c} d_{2}^{T}=\left[y_{0},y_{0},\ldots ,y_{0}\right]. \end{array}$$

Substituting (38), (40), and (42) into (1), we get

(44) KTϕt=ATϕ(t)−(μ+1)GαTϕ(t) +HβTϕ(t)Gϕ(t)ϕT(t)GαT,RTϕt=μGαTϕt−HβTϕtGαTϕtϕTtG.

Now using matrix of multiplication (36) in (44) we have

(45) $$\begin{array}{c} K^{T}\phi \left(t\right)=A^{T}\phi \left(t\right)-\left(\mu +1\right)G_{\alpha }^{T}\phi \left(t\right)+\phi^{T}\left(t\right)\hat{H}\hat{G}G,\\ R^{T}\phi \left(t\right)=\mu G_{\alpha }^{T}\phi \left(t\right)-\phi^{T}\left(t\right)\hat{H}\hat{G}G, \end{array}$$

which yields the system

(46) $$\begin{array}{c} \left(K^{T}-A^{T}+\left(\mu +1\right)G_{\alpha }^{T}-G_{\alpha }^{T}{\hat{G}}^{T}{\hat{H}}^{T}\right)\phi \left(t\right)=0,\\ \left(R^{T}-\mu G_{\alpha }^{T}+G_{\alpha }^{T}{\hat{G}}^{T}{\hat{H}}^{T}\right)\phi \left(t\right)=0. \end{array}$$

Using the independent property of Bernstein polynomials we obtain

(47) $$\begin{array}{c} K^{T}-A^{T}+\left(\mu +1\right)G_{\alpha }^{T}-G_{\alpha }^{T}{\hat{G}}^{T}{\hat{H}}^{T}=0,\\ R^{T}-\mu G_{\alpha }^{T}+G_{\alpha }^{T}{\hat{G}}^{T}{\hat{H}}^{T}=0. \end{array}$$

Solving this system for the vectors  *K*,  *R*,  we can approximate *x*(*t*) and *y*(*t*) from (40) and (42) respectively.

## 5. Illustrative Examples

Below we use the presented approach to solve two examples.

**Example 3 .**

We consider fractional-order Brusselator system given in [19] by

(48) $$\begin{array}{c} D_{t}^{\alpha }x\left(t\right)=-2x\left(t\right)+x{\left(t\right)}^{2}y\left(t\right),\\ D_{t}^{\beta }y\left(t\right)=x\left(t\right)-x{\left(t\right)}^{2}y\left(t\right), \end{array}$$

with initial conditions *x*(0) = 1 and *y*(0) = 1.

**Figure 1:**
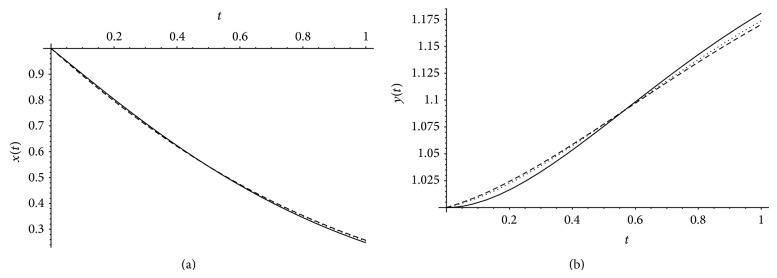
The exact solution (black line) and approximation solutions when *α* = 1, *β* = 1, and *m* = 12 (dotted) and *m* = 8 (dashed).

**Figure 2:**
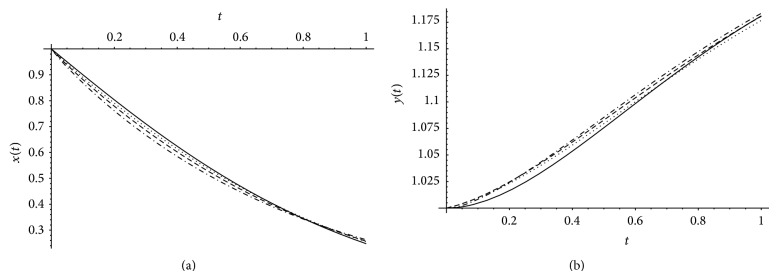
The exact solution (black line) and approximation solutions when *m* = 12 and *α* = .98, *β* = 1 (dotted), *α* = .95 and *β* = .99 (dashed), and *α* = .9 and *β* = .98 (Long-dashed).

Figure 1 presents comparison between exact solution and approximate solution obtained by the help of Bernstein polynomials for *x*(*t*), *y*(*t*) at *α* = 1, *β* = 1 when *m* = 8,12.  Figure 2 presents comparison between the exact solution and our approximate solution by Bernstein polynomials for *x*(*t*), *y*(*t*) at *m* = 12 and different values of *α* and *β*.

**Example 4 .**

We demonstrate accuracy of the presented numerical scheme by considering the fractional-order Brusselator system given in [19] by

(49) $$\begin{array}{c} D^{\alpha }x\left(t\right)=0.5-1.1x\left(t\right)+x{\left(t\right)}^{2}y\left(t\right),\\ D^{\beta }y\left(t\right)=0.1x\left(t\right)-x{\left(t\right)}^{2}y\left(t\right), \end{array}$$

with initial conditions *x*(0) = 0.4 and *y*(0) = 1.5.

**Figure 3:**
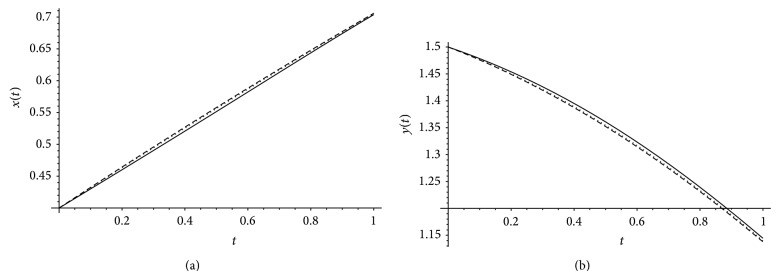
The exact solution (black line) and approximation solutions when *α* = 1, *β* = 1, and *m* = 6 (dotted) and *m* = 4 (dashed).

**Figure 4:**
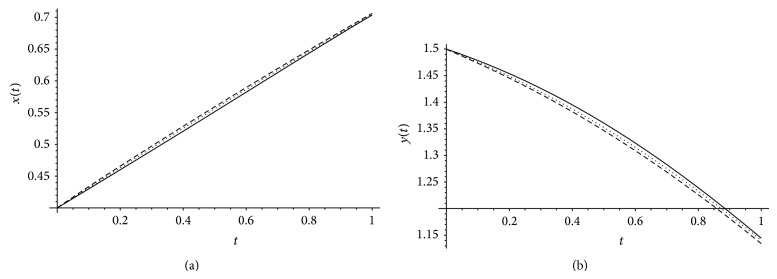
The exact solution (black line) and approximation solutions when *m* = 6 and *α* = 1, and *β* = .98 (dotted) and *α* = .98, *β* = .95 (dashed).

Figure 3 demonstrates the exact solution together with the approximate solutions *x*(*t*), *y*(*t*) for *α*, *β* = 1 and different values of *m* = 4,6. Definitely, by increasing the value of *m* of Bernstein basis, the approximate values of *x*(*t*), *y*(*t*) converge to the exact solutions. From the approximate solutions *x*(*t*), *y*(*t*) together with the exact solution for *m* = 6 and different values of *α*, *β* plotted in Figure 4 we see that as *α* approaches 1, the numerical solution converges to exact solution.

## 6. Conclusion

Due to the applications of fractional differential equations in the daily life of so many scientific disciplines as discussed in Section 1, we see many interesting results for its numerical solutions in the available literature as cited in the references via different mathematical tools. We have also been attracted towards the numerical solutions of fractional differential equations and have presented a numerical solution of the fractional-order Brusselator system given in (1) and (2) using the operational matrices of fractional integration and multiplication based on Bernstein polynomials. The proposed method is used due to the simplicity and accurateness in most of the cited work in which the fractional-order differential equations were expressed in the system of algebraic equations which were easily handled for their numerical solutions. For testing the accurateness of the scheme, we give two illustrative examples which show that the results are in agreement with the exact solutions. The numerical simulations were carried out using* Mathematica*.
